# Interactions Between Sedentary Behaviour, Moderate‐Vigorous Intensity Physical Activity and Acute Psychological Stress‐Induced Inflammatory Responses

**DOI:** 10.1002/smi.70038

**Published:** 2025-05-02

**Authors:** Victoria G. Linsley, Nicolette C. Bishop, Matthew J. Roberts, Malik Hamrouni, Mayada Demashkieh, Nicola J. Paine

**Affiliations:** ^1^ School of Sport, Exercise and Health Sciences National Centre for Sport and Exercise Medicine Loughborough University Loughborough UK; ^2^ National Institute for Health Research (NIHR) Leicester Biomedical Research Centre University Hospitals of Leicester NHS Trust and the University of Leicester Leicester UK

**Keywords:** active stress, inflammatory markers, passive stress, physical activity, psychological stress, sedentary behaviour

## Abstract

Psychological stress, physical activity (PA) and sedentary behaviour (SB) are modifiable risk factors for cardiovascular disease (CVD), possibly through altering one's inflammatory profile. The links between inflammatory responses to acute psychological stress and habitual moderate‐vigorous physical activity (MVPA) levels and SB volume is not clear. We explored the relationships in the magnitude of inflammatory responses to passive and active psychological stress with habitual MVPA and SB levels. Eighty‐eight healthy participants completed this study. Habitual MVPA and SB volume were measured over 1 week using wearable devices. The main trial consisted of a baseline period, a 6‐min passive (International Affective Picture System: IAPS) and an 8‐min active stress task (Paced Auditory Serial Addition Test: PASAT) with 45‐min rest post‐tasks. Heart rate (HR), systolic blood pressure (SBP) and diastolic blood pressure (DBP) were measured throughout the testing protocol. Blood samples were collected after each time point to measure circulating and lipopolysaccharide (LPS)‐stimulated interleukin‐6 (IL‐6), systemic inflammation response index (SIRI) and the neutrophil:lymphocyte ratio (NLR). There was a significant positive relationship between changes in HR during the IAPS and habitual SB (*B* = 1.061; *p* = 0.008). There were no relationships between the change in SBP or DBP during the IAPS and habitual SB (all *p* > 0.05). There were no relationships between acute psychological stress‐induced circulating IL‐6, LPS‐stimulated IL‐6, NLR or SIRI and habitual MVPA or SB levels. This is the first study to investigate passive psychological stress‐induced responses in the context of SB and builds on previous work in relation to SB and inflammatory responses to active stress. We found no associations between the inflammatory response to a passive or active psychological stress task and SB or MVPA levels in healthy young adults, but since our participants were very lean (21.7% body fat), findings may differ in other populations.

## 1 Introduction

Cardiovascular disease (CVD) continues to be a major health burden as globally, 1 in 13 people reportedly live with a form of CVD (British Heart Foundation 2024). Inflammation is a strong risk factor for CVDs and is the driver of each stage of atherosclerotic plaque development (Libby 2021). A plethora of risk factors have been associated with CVD development, including lifestyle and psychosocial factors, which may be linked by inflammatory mediators (Henein et al. 2022). Acute psychological stress is a risk factor for CVD, as it can trigger cardiac events as evidenced by a marked increase in cardiac episodes following natural disasters such as earthquakes (Gold et al. 2007), in response to emotive sporting events (Lin et al. 2019) and during emotional upset/anger (Buckley et al. 2015).

Acute psychological stress exposure activates the sympathetic nervous system and hypothalamic pituitary adrenal axis, leading to the release of catecholamines and cortisol to initiate and sustain physiological, immune and endocrine responses to return the body to a state of homeostasis (Dunlavey 2018; Steptoe and Brydon 2009). These responses may be adaptive when faced with a physical challenge or tissue damage/disease, however, in response to a psychological stressor, they appear metabolically unjust as they prime the body to cope with a physical threat which does not always ensue (Obrist 1976; J. R. Turner and Carroll 1985). Hence, repeated or exaggerated responses to acute psychological stress could lead to increased cardiovascular events through an increased inflammatory profile, for example, increased interleukin (IL)‐6 and tumour necrosis factor (TNF)‐α release which occurs up to 2 h post stress exposure (Hinterdobler et al. 2021; Marsland et al. 2017).

Other behavioural and lifestyle factors that are linked to CVD risk include physical activity (PA), which refers to any bodily movement, produced by skeletal muscles which results in an energy expenditure above that at rest (Caspersen et al. 1985). PA levels are associated with future CVD risk, with those engaging in greater weekly volumes of moderate‐vigorous intensity PA (MVPA) presenting with a lower risk of developing CVD (Ramakrishnan et al. 2021). Also, relationships between weekly PA engagement and lower stress related BP responses have been reported, suggesting that habitual PA could dampen the exaggerated cardiac reactivity observed in response to a psychological stressor (Thomas et al. 2019). Greater PA engagement has also been associated with enhanced parasympathetic tone and higher heart rate variability (Besnier et al. 2017), which may encourage a more efficient cardiovascular response to a psychological stressor (Chen et al. 2022). Though PA levels and acute psychological stress are independent risk factors for CVD, the relationship between these two entities and inflammatory/immune changes under stress remains unclear. One study reported a greater increase in lymphocyte numbers in response to acute stress in a ‘low’ self‐reported PA group compared to the ‘high’ PA group (Hong et al. 2004). However, associations between device‐measured PA and inflammatory responses to acute psychological stress, especially in response to passive forms of stress, have not been investigated.

Sedentary behaviour (SB) refers to any actions in a seated, reclining or lying position with an energy expenditure ≤ 1.5 metabolic equivalent of tasks, excluding sleep (Tremblay et al. 2017). Higher SB engagement has been associated with greater CVD incidence and mortality risk (Bailey et al. 2019; Patterson et al. 2018), independent of PA levels (Ekelund et al. 2019) and could therefore be considered as a separate entity in inflammatory disease risk. The research surrounding habitual SB volume and the inflammatory response to acute psychological stress is sparse. A systematic review (see Chauntry, Bishop, Hamer, and Paine (2022)) exploring the impact of habitual SB and acute psychological stress revealed only one paper assessing immune reactivity. PA was replaced with SB over a 2‐week period and circulating IL‐6 reactivity to active acute stressors was measured (Endrighi et al. 2016). No significant differences in IL‐6 reactivity between the conditions were reported but physical inactivity (i.e., lack of movement) was used as a proxy for SB (Endrighi et al. 2016). Recently, a study found a significant positive association between daily SB volume (inclinometer‐measured) and circulating IL‐6 levels 45‐min post‐acute active psychological stress exposure (Chauntry, Bishop, Hamer, Kingsnorth, et al. 2022). However, to our knowledge, there are no studies examining the potential associations between SB and other inflammatory changes to stress, and previous studies only utilised an active stress paradigm, highlighting the need for more research in this area in both passive and active stress tasks. Acute active (engaging in a task e.g., sitting an exam) and passive (enduring a task/situation e.g., financial or family worries/pressures) psychological stressors appear to perturb different pathways (Linsley et al. 2025; Marsland et al. 2002), yielding an inflammatory response. Therefore, habitual SB and MVPA volume may be related differently to these stress responses. To determine if behavioural interventions may be an effective way to dampen the stress‐induced increases in levels of inflammatory markers (e.g., circulating IL‐6 levels), it is first important to explore if there is a relationship between different forms of stress and the aforementioned behaviours to potentially target these in future studies. Therefore, this study aimed to incorporate both active and passive forms of acute psychological stress and explore the relationship between the stress‐induced increases in markers of inflammation and SB/MVPA volume.

Circulating IL‐6, and lipopolysaccharide (LPS) stimulated IL‐6 production are commonly assessed markers in the context of acute psychological stress reactivity (Marsland et al. 2017). Circulating IL‐6 is a strong predictor of future CVD development, including atherosclerosis (Okazaki et al. 2014) and coronary heart disease (Kaptoge et al. 2014). Stimulated IL‐6 levels may provide an insight into the innate immune response of activated immune cells which express toll‐like receptor‐4 (an LPS pattern recognition receptor) such as monocytes, to a greater extent than circulating levels (Yücel et al. 2017) as IL‐6 can be secreted from multiple sources (e.g., adipocytes). An exaggerated stimulated IL‐6 response may increase the risk of inflammatory conditions, but a dampened response may leave the individual susceptible to infection, which could be an important consideration when assessing this in response to acute psychological stress (Prather et al. 2009).

Novel leucocyte ratios have been developed including the neutrophil:lymphocyte ratio (NLR) and system inflammation response index (SIRI: [neutrophil counts × monocyte counts]/lymphocyte counts) as a biomarker of inflammation (Marchi et al. 2024). These markers may predict future CVD risk more accurately than using individual leucocyte counts and provide a relatively low‐cost option to identify individuals at risk of developing CVD (Xia et al. 2023). Higher SIRI levels have been associated with greater CVD risk, in a dose‐dependent manner (Jin et al. 2021). Additionally, higher NLR levels were associated with a greater risk of developing CVDs (Angkananard et al. 2018) and increased depression risk (Cheng et al. 2022). Significant passive and active psychological stress‐induced increases in SIRI have been reported, whereas NLR increases were only significant in response to an active stressor (Linsley et al. 2025). However, the impact of MVPA and SB on NLR and SIRI reactivity to acute psychological stress remains unclear.

Therefore, in this study we aimed to elucidate any relationships between habitual MVPA and SB levels with circulating IL‐6, LPS‐stimulated IL‐6 production, SIRI and NLR reactivity to both a passive and active psychological stressor. It was hypothesised that (1) higher engagement in regular MVPA would be significantly related to a smaller increase in these inflammatory markers, in response to an acute active psychological stressor but no significant association with a passive stressor and; (2) higher habitual SB engagement would be significantly associated with a less favourable inflammatory response magnitude, such as higher circulating IL‐6 levels, in response to an acute active psychological stressor but no significant relationship with the passive stress task.

## 2 Methods

### 2.1 Participants

A sample of 92 healthy, young (aged 18–25 years) individuals enrolled in this study during the period of March 2022 to June 2023, as part of a wider study, data from which has been reported elsewhere (Linsley et al. 2025). Participants were excluded if: (1) BMI was > 40 kg/m^2^; (2) resting brachial systolic blood pressure (SBP) was > 140 mmHg; (3) they had a current or previous clinically diagnosed psychological condition; (4) they had a clinically diagnosed disease/condition; (5) they had a history of or currently smoked/used recreational drugs (including e‐cigarettes and vapes) or (6) they were taking prescribed or over‐the‐counter medication which could affect the outcomes of interest (e.g., anti‐inflammatory drugs). Before the stress‐reactivity trial, participants avoided: alcohol for 12 h; vigorous exercise for 24 h; food and drink (other than water) for 4 h and over‐the‐counter medication for 72 h. The visit was rescheduled if the participant was ill/infectious (self‐reported) or had been in the previous 7 days. This study was conducted in accordance with the Code of Ethics of the World Medical Association (Declaration of Helsinki). All participants gave informed consent and ethical approval was given by the Loughborough University ethics committee (2021‐5456‐4541).

### 2.2 Protocol

This study consisted of two visits. The first screening visit included obtaining written informed consent from the participant, a resting brachial blood pressure measurement (Omron M6 Comfort, Omron Healthcare, Milton Keynes, UK) and completion of a series of validated questionnaires to ensure the participant met the inclusion criteria and to collect socio‐demographic and anxiety (General Anxiety Disorder‐7 [GAD7]) and depressive (Hospital Anxiety Depressive Scale‐Depression Score [HADS‐D]) symptom information, since anxiety and depressive symptoms have been associated with higher levels of inflammatory markers (e.g., circulating IL‐6) (Meyer et al. 2011; Pitsavos et al. 2006). The GAD7 is a 7‐item questionnaire related to general anxiety symptoms (Spitzer et al. 2006), which is widely used in clinical and research settings due to its reliability and validity (Dear et al. 2011; Toussaint et al. 2020). HADS‐D has been shown to be valid in assessing depressive symptoms in the general population (Bjelland et al. 2002). The GAD‐7 questionnaire had ‘good’ internal consistency (*α* = 0.8) and the HADS‐D Cronbach's alpha was = 0.5. Two activity monitoring devices were provided to the participant to wear for the next 8 days, to estimate time spent in SB (ActivPAL3 inclinometer; PAL Technologies Ltd, Glasgow, UK) and daily PA levels (Actigraph GT3X BT+, ActiGraph, Florida, USA).

After at least 8 days, participants returned to the laboratory for the main trial (Figure S1). This consisted of a 20‐min baseline period of rest, one active stress task, one passive stress task and two 45‐min recovery periods. The order in which the stress tasks took place was randomly assigned to each participant. Every main trial started at 10 AM to avoid any diurnal changes in outcome measures. Height (274 stadiometer, Seca GmbH, Hamburg, Germany), weight, body fat percentage (mBCA 515 bioimpedence scales, Seca GmbH, Hamburg, Germany), waist circumference (Seca 201 tape measure, GmbH, Hamburg, Germany) and resting brachial blood pressure (Omron M6 Comfort, Omron Healthcare, Milton Keynes, UK) were measured.

The participant was seated throughout the session. A nature documentary (Frozen Planet, BBC, UK) was played during the rest periods (Chauntry, Bishop, Hamer, Kingsnorth, et al. 2022; Paine et al. 2013, 2014). The Borg 6–20 rating of perceived exertion scale (Borg 1982) and a 7‐point Likert scale (rating of 0–6; 0 being ‘not at all’, 6 being ‘extremely’) were used at each time point to assess how difficult, stressful, arousing, engaging and how well the participant thought they were doing.

### 2.3 SB Measurement

A 20 Hz ActivPAL3 inclinometer (PAL Technologies Ltd, Glasgow, UK) was used to determine SB, as it uses an ‘Intelligent Activity Classification’ algorithm and accelerometer to determine limb position to infer time spent in seated, lying and upright positions. The device was inserted into a waterproof nitrile sleeve and secured to the upper anterior thigh of the non‐dominant leg with Hypafix roll (BSN Medical, Hamburg, Germany), which has been described in more detail elsewhere (Chauntry, Bishop, Hamer, Kingsnorth, et al. 2022). Briefly, participants wore the monitor at all times, unless it would be submerged in water (e.g., during water sports). If the device was removed, extra stickers were provided and support from the research team was given to reapply the monitor. SB was classified as a seated or lying posture (thigh angle < 20° difference from the horizontal plane) with a MET value of < 1.25. To be included in the analysis, at least 4 valid days (≥ 1 weekend day and ≥ 3 weekdays) were required; a valid day was defined as > 499 steps, < 95% of time in any one posture and ≥ 10 h wear time (Edwardson et al. 2017). The data were downloaded using the validated ActivPAL3 computer software (PAL Technologies Ltd, Glasgow, UK) and analysed via ProcessingPAL (PAL Technologies Ltd, Glasgow, UK). Participants completed a daily wear time, activity and sleep log which was used to cross‐check the device measured activities. The data was also screened using heat maps to check for any misclassifications in the algorithm‐derived output. Any clear errors were manually corrected (Edwardson et al. 2017).

### 2.4 PA Measurement

A triaxial accelerometer (ActiGraph GT3X BT+, ActiGraph, Florida, USA) was worn on the non‐dominant wrist to obtain data on time spent in different intensities of PA over a 7‐day period. The device was worn during sleep but removed for water‐based activities (e.g., bathing or swimming), as detailed elsewhere (Chauntry, Bishop, Hamer, Kingsnorth, et al. 2022). At least 4 valid days (consisting of at least 16 h of wear time) of data were required (≥ 3 weekdays and ≥ 1 weekend day) to be included in the analysis (Chauntry, Bishop, Hamer, Kingsnorth, et al. 2022; Mikkelsen et al. 2020). Devices were set to record at a sampling frequency of 100 Hz using the ActiLife software (v6.13.4, Actigraph, Florida, USA), and analysed with GGIR (an open‐source R package, version 3.1‐5) (Migueles et al. 2019). PA was defined as follows: inactivity < 44.8 mg; light PA 44.8 to < 100.6 mg; moderate PA 100.6 to < 428.8 mg; vigorous PA > 428.8 mg (Hildebrand et al. 2014, 2017).

### 2.5 Active Stress Task: Paced Auditory Serial Addition Test (PASAT)

The PASAT (8‐min version (Gronwall 1977)) was used as an active stressor. The task involved adding two numbers (between 1 and 9) read out by an audio file, remembering the second digit and adding it to the next number read out. The time between number presentations reduced as the test proceeded (2.4, 2.0, 1.6, 1.2 s, respectively (Tombaugh 2006)). Prior to starting the test, an instruction tape was played through a tablet device (iPad, Apple Inc., California, USA) followed by a practice test, then the main task commenced. To enhance the stressfulness of the task, elements of social evaluation and competition were included, which have been described elsewhere (Paine et al. 2013, 2014). A researcher stood close to the participant with a clipboard to mark the test. A deterring buzzer was sounded every 10 numbers, in response to an incorrect answer or hesitation. If no mistake was made, the buzzer was pressed on the 10th number to maintain consistency on the number of aversive sounds given to each participant (Paine et al. 2014).

### 2.6 Passive Stress Task: International Affective Picture System (IAPS)

The IAPS was used as a passive stressor, which consisted of a bank of standardised images to induce an emotional response (Lang et al. 1997). Images from the ‘negative valence’ group were selected and displayed to evoke a stress response. The task lasted 6‐min and the participants were asked to look at the screen throughout. If at any point they looked away from the screen, they were asked to refocus.

### 2.7 Cardiovascular Measures

Over a 10‐min baseline period, throughout each stress task and for 8‐min immediately after each stress task, continuous cardiovascular measures were collected. A human non‐invasive blood pressure (NIBP) system (ADInstruments, Oxford, UK) was used to measure SBP and diastolic blood pressure (DBP) by putting an oscillometric blood pressure cuff on the middle phalanx of the middle finger to identify changes in arterial diameter. Lab Chart 8 (ADInstruments, Oxford, UK) was used for data collection and analysis. A 3‐lead electrocardiogram (ECG), with electrodes placed below each clavicle and the lower left rib, was used to measure heart rate (HR). The ECG signal was amplified (PowerLab, ADInstruments, Oxford, UK) and analysed using the Lab Chart 8 software program (ADInstruments, Oxford, UK).

### 2.8 Blood Sampling and Analysis

A 20‐gauge cannula (BD Nexiva, BD, New Jersey, USA) was inserted into a suitable antecubital vein to allow blood draws to be made at each time point (baseline, immediately post‐stress and 45‐min post‐stress tasks). The first 2 mL of blood was drawn into a syringe and disposed of, with 4.9 mL of blood collected in a Potassium Ethylene Diamine Tetra Acetic Acid (K3 EDTA) S‐monovette tube (Sarstedt AG & Co. KG, Nümbrecht, Germany) and two 7.5 mL samples collected in S‐monovette tubes treated with sodium heparin (Sarstedt AG & Co. KG, Nümbrecht, Germany). After each collection, the cannula was flushed with a 0.9% NaCl solution.

At each time point, total and differential leucocyte counts (neutrophils, lymphocytes and monocytes) were measured using 20 μL of EDTA blood on a haematology analyser (Yumizen H500, Horiba Medical, Montpellier, France). A neutrophil to lymphocyte ratio (NLR) was calculated (neutrophil count/lymphocyte count) and a system inflammation response index (SIRI) was also assessed ([neutrophil count × monocyte count]/lymphocyte count]). The remaining blood was centrifuged (3500 rpm, 10 min, 4°C) before the plasma was aliquoted (∼500 μL) into sterile micro tubes and stored at −80°C for future analysis. Circulating IL‐6 was subsequently measured in duplicate using high‐sensitivity ELISA kits (R&D Systems, Minneapolis, USA), according to manufacturer's instructions. The intra‐assay coefficient of variance was 2.0%.

PBMCs were isolated from whole blood (treated with sodium heparin) using the density‐gradient centrifugation technique for each time point. After isolating PBMCs, the cells were counted using a haemocytometer (BRAND GMBH + CO KG; Wertheim, Germany) and 250 μL of the cell suspension was seeded onto a 24‐well plate at a concentration of 170,000 cells/μL in media (RPMI). The cells were either stimulated with 25 μL LPS (500× dilution in RPMI) with the addition of 225 μL RPMI or 250 μL RPMI was added to the cell suspension to be used as a non‐stimulation control condition (each well contained a total volume of 500 μL). After a 4‐h incubation period (CO_2_: 5%, temperature: 37°C), the supernatant from each well was centrifuged (300 RCF, 5 min) and the resultant supernatant was aliquoted (∼200 μL) into two microtubes and stored at −80°C for future analysis. LPS‐stimulated IL‐6 production from PBMCs was assessed in duplicate using ELISA kits (R&D Systems, Minneapolis, USA), according to manufacturer's instructions. The intra‐assay coefficient of variance was 4.8%.

### 2.9 Data Reduction and Statistical Analysis

Data were analysed using IBM SPSS Statistics version 29 (IBM, Chicago, USA), with statistical significance was set at *p <* 0.05. Missing Actigraph, ActivPAL and inflammatory marker data was imputed using a 5× multiple imputation method (Little and Rubin 2003). Normality tests were run on each dependent variable and Kolmogorov–Smirnov significance of < 0.05 signified data was non‐normally distributed. Generalised linear models (GLM) explored relationships between stress‐induced changes in markers of inflammation/cardiovascular measures and habitual MVPA/SB levels. The change in inflammatory/cardiovascular measures was calculated as ‘baseline’ value minus ‘time point’ value. As all inflammatory markers were non‐normally distributed, gamma with log link models were used. HR and SBP were normally distributed so linear models were used, DBP was non‐normally distributed, hence, gamma with log link models were applied. Unstandardised *B*, 95% Wald confidence intervals and *p* values are presented. GLM analyses were adjusted for the following covariates in line with previous research in this field (Chauntry, Bishop, Hamer, Kingsnorth, et al. 2022): age, biological sex (male or female), body fat percentage, ethnicity, task order, MVPA volume (for SB models)/light PA volume (for MVPA models), device wear time, GAD‐7 score, HADS‐D score and baseline inflammatory/cardiovascular measures. Time‐by‐group interactions were assessed using generalised estimating equations (GEE). Four groups were formed based on below (low)/above (high) mean MVPA and SB: (1) high MVPA, high SB; (2) high MVPA, low SB; (3) low MVPA, high SB and (4) low MVPA, low SB. GEEs were adjusted for age, biological sex (male or female), ethnicity, task order, body fat percentage, device wear time, GAD‐7 score and HADS‐D score. Wald chi‐square, *p*‐values and Cramer's *V* effect sizes (degrees of freedom = 3, therefore, *V* = 0.06 [small effect]; *V* = 0.17 [medium effect]; *V* = 0.29 [large effect]) are presented.

#### 2.9.1 Sample Size Determination

The sample size was determined using an effect size based on previous literature investigating the relationships between circulating IL‐6 changes in response to acute psychological stress and measures of SB (Chauntry, Bishop, Hamer, Kingsnorth, et al. 2022). Using RStudio (RStudio 2023.03.0 + 386) (pwrss package) with an effect size of *f* ^2^ = 0.02, power = 0.8, *α* = 0.05, and accounting for inclusion of covariates, the minimum sample size was determined to be 79.

## 3 Results

### 3.1 Participant Characteristics

Eighty‐eight participants completed both study visits. Table 1 provides an overview of the participant characteristics. The mean age of participants was 21.5 (SD = 2.2) years, 41% of the sample were female and 51% were of white ethnicity. Mean BMI was 23.6 (3.2) kg/m^2^ and mean body fat % was 21.7 (7.6)%. Average daily engagement in SB was 9.4 (SD = 1.5) h/day and mean MVPA levels were 97.3 (SD = 29.6) min/day. There were significant changes in circulating IL‐6, LPS‐stimulated IL‐6, SIRI and NLR in response to stress, which have been reported in more detail elsewhere (Linsley et al. 2025).

**TABLE 1:** Participant characteristics at baseline.

Variable	Mean (SD)/*N* (%)
Baseline inflammatory markers	
Circulating IL‐6 (pg/mL)	0.77 (0.59)
Leucocyte counts (×10^9^/L)	5.15 (1.17)
Monocyte counts (×10^9^/L)	0.41 (0.11)
Neutrophil counts (×10^9^/L)	2.78 (0.85)
Lymphocyte counts (×10^9^/L)	1.70 (0.42)
NLR	1.73 (0.64)
SIRI	0.72 (0.40)
LPS‐stimulated IL‐6 (pg/mL)	43.2 (22.0)
ActivPAL data	
Daily SB (h/day)	9.4 (1.5)
Waking wear time (h/day)	15.0 (0.9)
Actigraph data	
Daily MVPA (min/day)	97.3 (29.6)
Daily LPA (min/day)	125.8 (39.7)
Waking wear time (min/day)	981.6 (80.7)

Abbreviations: IL‐6 = interleukin‐6; LPA = light intensity physical activity; LPS = lipopolysaccharide; MVPA = moderate‐vigorous intensity physical activity; NLR = neutrophil‐lymphocyte ratio; SB = sedentary behaviour; SIRI = system inflammation response index.

### 3.2 Relationship Between SB, MVPA and Cardiovascular Responses to Acute Psychological Stress

There was a significant positive relationship between the change in HR during the IAPS (baseline to IAPS) and habitual SB (*B* = 1.067; 95% CI = 0.293, 1.840; *p* = 0.007). There were no significant relationships between the change in SBP or DBP during the IAPS and habitual SB (all *p* > 0.05). No significant relationships existed between PASAT‐induced changes in cardiovascular measures and habitual SB volume (all *p* > 0.05). There were no significant relationships between PASAT‐induced or IAPS‐induced changes in any cardiovascular measures and habitual MVPA (all *p* > 0.05).

### 3.3 Relationship Between SB, MVPA and Immune Responses to Acute Psychological Stress

As shown in Table 2, in response to the passive stress task, there were no significant relationships between IAPS‐induced changes in circulating IL‐6 levels, LPS‐stimulated IL‐6 levels, NLR or SIRI (all *p* > 0.05) and habitual SB levels. In response to the active stress task, there were no significant relationships between PASAT‐induced changes in any of the measured markers of inflammation and habitual SB levels (all *p* > 0.05). There were no significant relationships between IAPS‐induced changes in any of the measured markers of inflammation and habitual MVPA volume (all *p* > 0.05; Table 3). There were no significant relationships between PASAT‐induced changes in any of the measured markers of inflammation and habitual MVPA volume (all *p* > 0.05).

**TABLE 2:** Relationship between the stress‐induced changes in inflammatory and cardiovascular markers and habitual sedentary time.

	*B*	SE	95% CI		*p*
			Lower	Upper	
BASELINE‐PASAT					
Δ circulating IL‐6	0.035	0.028	−0.021	0.091	0.217
Δ LPS‐stimulated IL‐6	−0.006	0.020	−0.046	0.034	0.766
Δ SIRI	−0.000	0.001	−0.001	0.001	0.534
Δ NLR	−0.001	0.001	−0.002	0.001	0.348
Δ HR	1.590	0.918	−0.209	3.390	0.083
Δ SBP	1.914	1.662	−1.343	1.327	0.249
Δ DBP	0.001	0.014	−0.027	0.028	0.961
BASELINE‐45‐min post PASAT					
Δ circulating IL‐6	0.054	0.032	−0.008	0.116	0.090
Δ LPS‐stimulated IL‐6	0.012	0.017	−0.020	0.045	0.466
Δ SIRI	−0.001	0.001	−0.003	0.002	0.512
Δ NLR	−0.001	0.002	−0.005	0.003	0.550
BASELINE‐IAPS					
Δ circulating IL‐6	0.021	0.026	−0.030	0.071	0.423
Δ LPS‐stimulated IL‐6	0.001	0.019	−0.036	0.038	0.952
Δ SIRI	−0.001	0.000	−0.001	0.000	0.181
Δ NLR	−0.000	0.001	−0.001	0.001	0.951
Δ HR	1.067	0.395	0.293	1.840	**0.007**
Δ SBP	−1.468	1.434	−4.278	1.342	0.306
Δ DBP	−0.004	0.011	−0.025	0.017	0.716
BASELINE‐45‐min post IAPS					
Δ circulating IL‐6	0.013	0.041	−0.068	0.094	0.760
Δ LPS‐stimulated IL‐6	−0.011	0.017	−0.043	0.022	0.521
Δ SIRI	−0.000	0.000	−0.001	0.000	0.268
Δ NLR	−0.001	0.000	−0.002	0.000	0.118

*Note:* Adjusted for: age, sex, body fat percentage, ethnicity, task order, GAD‐7 score, HADS‐D score, ActivPAL wear time, MVPA and baseline immune marker levels. Bold indicates statistical significance (*p* < 0.05).

Abbreviations: DBP = diastolic blood pressure; HR = heart rate; IAPS = international affective picture system; IL‐6 = interleukin‐6; LPS = lipopolysaccharide; NLR = neutrophil lymphocyte ratio; PASAT = paced auditory serial addition test; SBP = systolic blood pressure; SIRI = system inflammation response index.

**TABLE 3:** Relationship between the stress‐induced changes in inflammatory and cardiovascular markers and habitual MVPA.

	*B*	SE	95% CI		*p*
			Lower	Upper	
BASELINE‐PASAT					
Δ circulating IL‐6	0.001	0.001	−0.001	0.003	0.312
Δ LPS‐stimulated IL‐6	−0.001	0.001	−0.002	0.001	0.363
Δ SIRI	0.000	0.000	−0.000	0.000	0.475
Δ NLR	0.000	0.000	−0.000	0.000	0.297
Δ HR	−0.001	0.034	−0.068	0.065	0.968
Δ SBP	−0.087	0.066	−0.215	0.042	0.186
Δ DBP	−0.001	0.001	−0.002	0.000	0.128
BASELINE‐45‐min post PASAT					
Δ circulating IL‐6	0.001	0.001	−0.001	0.004	0.389
Δ LPS‐stimulated IL‐6	−0.000	0.001	−0.001	0.001	0.580
Δ SIRI	0.000	0.000	−0.000	0.000	0.308
Δ NLR	0.000	0.000	−0.000	0.000	0.202
BASELINE‐IAPS					
Δ circulating IL‐6	0.001	0.001	−0.001	0.003	0.218
Δ LPS‐stimulated IL‐6	−0.001	0.001	−0.002	0.001	0.457
Δ SIRI	0.000	0.000	−0.000	0.000	0.740
Δ NLR	0.000	0.000	−0.000	0.000	0.255
Δ HR	0.004	0.016	−0.028	0.036	0.799
Δ SBP	−0.042	0.057	−0.154	0.070	0.464
Δ DBP	−0.000	0.000	−0.001	0.000	0.426
BASELINE‐45‐min post IAPS					
Δ circulating IL‐6	0.001	0.002	−0.002	0.004	0.670
Δ LPS‐stimulated IL‐6	−0.000	0.001	−0.001	0.001	0.970
Δ SIRI	0.000	0.000	−0.000	0.000	0.340
Δ NLR	0.000	0.000	−0.000	0.000	0.170

*Note:* Adjusted for: age, sex, body fat percentage, task order, GAD‐7 score, HADS‐D score, ethnicity, Actigraph wear time, Light PA volume, baseline inflammatory marker levels.

Abbreviations: DBP = diastolic blood pressure; HR = heart rate; IAPS = international affective picture system; IL‐6 = interleukin‐6; LPS = lipopolysaccharide; MVPA = moderate‐vigorous intensity physical activity; NLR = neutrophil:lymphocyte ratio; PASAT = paced auditory serial addition test; SBP = systolic blood pressure; SIRI = system inflammation response index.

### 3.4 Interaction Between MVPA and SB Volume With Immune Responses to Acute Psychological Stress

**FIGURE 1:**
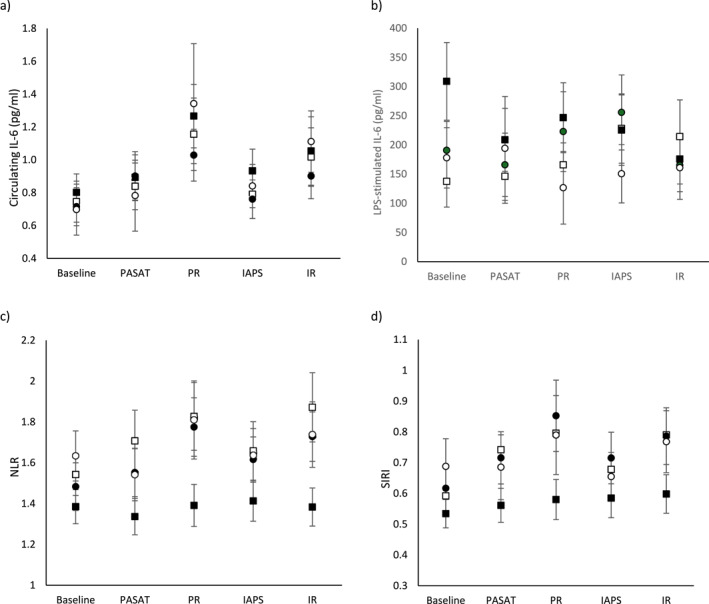
Group by time effects on (a) circulating IL‐6 levels; (b) LPS‐stimulated IL‐6 levels; (c) NLR; (d) SIRI. Closed circle = low MVPA, high SB; closed square = high MVPA, low SB; open circle = low MVPA, low SB; open square = high MVPA, high SB. PASAT and IAPS were completed in a randomised order. IL‐6 = interleukin‐6; LPS = lipopolysaccharide; MVPA = moderate‐vigorous intensity physical activity; NLR = neutrophil:lymphocyte ratio; SB = sedentary behaviour; SIRI = systemic inflammation response index ([neutrophil count × monocyte count]/lymphocyte count]). Data is presented as mean ± standard error.

Participants were grouped based on habitual MVPA and SB levels, yielding 16 individuals in the high MVPA, high SB group; 27 in the high MVPA, low SB group; 28 in the low MVPA, high SB group and 17 in the low MVPA, low SB group. GEE models (Figure 1) revealed a significant overall group‐by‐time interaction for NLR (*χ* ^2^ = 27.62; *p* = 0.006; *V* = 0.32) and SIRI (*χ* ^2^ = 29.64; *p* = 0.003; *V* = 0.34) but no significant post‐hoc differences were observed. There were no significant group‐by‐time interaction effects for circulating IL‐6 (*χ* ^2^ = 20.01; *p* = 0.067; *V* = 0.28) or LPS‐stimulated IL‐6 (*χ* ^2^ = 17.36; *p* = 0.137; *V* = 0.26).

## 4 Discussion

Our study explored associations between habitual MVPA and SB volume and inflammatory changes to passive and active acute psychological stress. This is the first study to look at interactions between MVPA/SB and inflammatory responses to passive psychological stress. Acute psychological stress, PA levels and SB volume are all independent risk factors for CVD, possibly via altering one's inflammatory profile, but the relationship between these factors remains unclear (Bays et al. 2022; Jingjie et al. 2022; Vancheri et al. 2022).

We found a significant positive relationship between the increase in HR during the IAPS and habitual SB volume. This is important as exaggerated HR reactivity has been shown to predict future CVD risk, including increased risk of sudden coronary death (A. I. Turner et al. 2020) and may highlight a potential mechanism behind how SB could increase CVD risk through acute psychological stress exposure. This relationship was only observed in response to a passive stressor, not an active psychological stressor, which is in agreement with a study that observed HR reactivity to an active stressor but found no relationship with habitual SB volume (Chauntry, Bishop, Hamer, Kingsnorth, et al. 2022). However, they did report a significant positive relationship between DBP reactivity and habitual SB volume, which is in contrast to our findings (Chauntry, Bishop, Hamer, Kingsnorth, et al. 2022). A systematic review also reported mixed results in terms of HR responses to acute psychological stress and SB volume, however, as noted by the authors, no studies measured SB via a postural component and therefore used physical inactivity as a proxy measure (Chauntry, Bishop, Hamer, and Paine 2022). Nevertheless, half of the studies (2/4 papers) found that physical inactivity levels (used as a proxy for SB) were associated with elevated HR reactivity to acute psychological stress, with the other half reporting no significant difference (Chauntry, Bishop, Hamer, and Paine 2022). All four of the included studies utilised an active stress task—ours is the first study to our knowledge to have investigated SB in the context of passive stress.

A multitude of factors may cause this increased HR response to acute psychological stress in those who are habitually more sedentary, but the mechanisms which underlie our findings must remain speculative. One possibility is that as greater SB volume has been associated with greater HR at rest, and while the clinical significance appears to be small (Alansare et al. 2021), other physiological changes such as blood pooling may occur with prolonged sedentary time, potentially leading to an increase in sympathetic nervous system (SNS) activity to increase HR and restore sufficient blood flow dynamics (Alansare et al. 2021; Restaino et al. 2015). However, since we did not directly measure SNS activity, this area requires more research, as the high occurrence of stress in everyday life could yield the potential for deleterious health consequences.

We found no relationships between SB volume and the inflammatory response (circulating IL‐6, LPS‐stimulated IL‐6, SIRI nor NLR) to an active or passive psychological stressor. We used the ‘gold‐standard’ ActivPAL device to quantify sedentary time using measures of acceleration and inclination (Sellers et al. 2016), but found no association with the magnitude of the inflammatory response. Our findings contrast others which also assessed SB using the ActivPAL device and reported a significant positive relationship between the change in circulating IL‐6 levels from baseline to 45‐min post‐PASAT and habitual SB volume (Chauntry, Bishop, Hamer, Kingsnorth, et al. 2022). We used the same active stressor, and the recorded mean SB volume was also similar between the two samples and representative of the UK population (average UK male = 9.5 h/day; females = 9.0 h/day) (Hamer et al. 2020). Therefore, our different findings could be due to our population being younger (mean age = 21.5 years; Chauntry et al., mean age = 25.69 years) or having a lower body fat percentage (mean = 21.7%; Chauntry et al. mean = 25.47%) (Chauntry, Bishop, Hamer, Kingsnorth, et al. 2022). IL‐6 is produced and secreted by numerous cells and tissues but white adipose tissue contributes up to 35% of resting circulating IL‐6 levels in humans and therefore, the lower body fat percentage in our study may explain the discrepancy in results (Mohamed‐Ali et al. 1997). However, both studies adjusted for body fat percentage in the analyses so further research is required to gain clarity on the relationship between SB volume and the inflammatory response to acute psychological stressors. Similarly, we are the first study to explore passive psychological stress in the context of inflammation and habitual SB volume so although we found no significant relationships, in other populations (such as those with obesity), the circulating IL‐6 reactivity to a passive stressor and SB relationship may be different as associations between SB volume and CVD risk are stronger in those who are physically inactive, hence, psychological stress‐induced circulating IL‐6 responses may also differ in a sample of physically inactive individuals (Katzmarzyk et al. 2019).

We did not find any associations between psychological stress‐induced inflammatory responses (circulating IL‐6, LPS‐stimulated IL‐6, NLR and SIRI) and MVPA volume. Our population was highly active with the mean MVPA being 97.3 min/day and the lowest recorded volume being 30.8 min/day, which is still around 1 h greater than the UK weekly PA guidelines. We used a wearable wrist‐worn device to estimate daily MVPA engagement, so higher values are expected, and our data is comparable to other studies in this field who have measured MVPA using wrist‐worn methodologies (Chauntry, Bishop, Hamer, Kingsnorth, et al. 2022). One study reported that higher MVPA levels, measured using a wrist‐based accelerometer, may prevent aortic valve stenosis but the median MVPA was notably lower than in our study (167 min/week (Li et al. 2024)). Since, a lack of PA (not meeting the PA guidelines) is a strong risk factor for CVD progression and mortality, the high volume of MVPA in our sample may have provided protection against the potentially deleterious inflammatory responses to acute psychological stress (Cabanas‐Sánchez et al. 2024). Future research should explore this relationship in a less active sample.

This is the first study to look at leucocyte count ratios in the context of acute psychological stress reactivity and MVPA volume. We found no significant relationships between NLR or SIRI reactivity and habitual MVPA levels. One study reported a dampened circulating lymphocyte response to a speech stress task in those who were classified as ‘highly physically active’ compared to those in the ‘low PA’ group (Hong et al. 2004). However, they used self‐reported questionnaire data making it difficult to compare the findings to our device‐measured PA data, since self‐reported PA has been shown to not correlate well with device‐measured data (Seo et al. 2021). Also, NLR and SIRI encompass cells from both the innate and adaptive immune system, potentially reflecting the systemic inflammatory response to a greater extent than looking at individual cell counts (Song et al. 2021). Aside from these two studies, habitual MVPA volume has not been explored as a potential moderator of the inflammatory response (i.e., leucocyte counts) to acute psychological stress so although our study advances the literature, future research is required.

We also examined IL‐6 levels released from PBMCs exposed to LPS and found no significant relationship between the stress‐induced change in LPS‐stimulated IL‐6 levels and habitual MVPA engagement. This is surprising since those who regularly exercise have been shown to exhibit a greater pro‐inflammatory cytokine response to a microbial antigen stimulation, via increased toll‐like receptor expression on PBMCs (Zheng et al. 2015), suggesting a more effective immune response to fight invading pathogens than those who do not regularly exercise. Our findings suggest that habitual MVPA levels do not impact the inflammatory response to a combination of acute psychological stress (either active or passive) and the threat of an invading pathogen. This may be deleterious in terms of infectious disease development when facing psychological stress in daily life, regardless of time spent engaging in MVPA, which highlights the holistic care required to maintain health and wellbeing.

We explored the impact of habitual SB and MVPA engagement on inflammatory responses to acute psychological stress. Despite a significant interaction effect for SIRI and NLR, no post‐hoc differences were evident. It is difficult to elucidate the relevance in terms of identifying combinations of SB and MVPA which may be beneficial for minimising an exaggerated inflammatory response to psychological stress exposure. This may have been due to differences in the number of participants in each group, and while we used mean MVPA (97.3 min/day) to determine ‘high’ and ‘low’ MVPA, values below this cut‐point may still be regarded as ‘high’ in the general population as almost 1/3 of the global population do not meet the PA guidelines (Strain et al. 2024). Since high MVPA engagement has been associated with protecting against the increased risk of CVD with increased time spent sitting (Ekelund et al. 2019), this may be why we did not see any post‐hoc differences for the inflammatory markers at any time points, despite sedentary time being high in our population (average SB = 9.4 h/day). Future research could explore this relationship in a less active cohort.

A main strength of this study included utilising both an active and a passive stressor to explore the relationship between acute psychological stress exposure and habitual SB/MVPA levels. We measured SB volume using the gold‐standard device, capable of detecting postural components that are a fundamental determinant of SB (Tremblay et al. 2017), used a device‐based measurement of MVPA and explored multiple inflammatory markers. However, there are some limitations to our work. We measured inflammatory markers immediately after the stress had subsided and 45‐min later, but this may not have been optimal to capture the peak response for circulating and LPS‐stimulated IL‐6 (Marsland et al. 2017). However, as a significant increase in circulating IL‐6 levels 45‐min after the PASAT had been observed previously (Chauntry, Bishop, Hamer, Kingsnorth, et al. 2022), our findings may not be attributable to the time of assessment. Due to participant burden, committing to 4 h of sitting in the laboratory, it may not have been feasible to take samples at later time points, or multiple samples during a recovery phase post‐stress, over a 4h fasted period. Additionally, given our sample consisted of young, healthy and highly physically active individuals (to reduce the risk of comorbidities impacting our findings), this potentially limits the transferability of these findings to other population groups. Other measures, including heart rate variability, should also be considered in future studies. It should also be noted that we used ‘negative valence’ images from the IAPS collection, whereas images promoting ‘positive’ emotions may lead to favourable inflammatory profile responses such as lower circulating IL‐6 levels (Stellar et al. 2015).

This study explored the impact of habitual SB and MVPA volume on changes in circulating IL‐6 levels, LPS‐stimulated IL‐6 levels, SIRI and NLR reactivity to acute active and passive psychological stress. Our study is the first to investigate these responses in the context of passive stress and builds on previous work in relation to SB and inflammatory responses to active stress. While we found no significant associations between the inflammatory response to a passive or active psychological stress task and SB or MVPA levels in healthy adults, our data provides important insights into the potential interactions between SB, MVPA and inflammation which could underlie stress‐induced risk of CVD.

## Conflicts of Interest

The authors declare no conflicts of interest.

## Supporting information

Figure S1

## Data Availability

The data that support the findings of this study are available from the corresponding author upon reasonable request, in line with ethical and consent considerations.
