# Supplementary material for: Interactions Between Sedentary Behaviour, Moderate‐Vigorous Intensity Physical Activity and Acute Psychological Stress‐Induced Inflammatory Responses
Source: Stress Health. 2025 May 2;41(3):e70038. doi: 10.1002/smi.70038 (PMC12047615; doi:10.1002/smi.70038)
Supplement: Supplementary file 1 — Figure S1 [file SMI-41-e70038-s001.docx]

**Supplementary Figure 1.** A schematic outline of the study protocol.

**Visit 1:**

**Screening**

**Visit 2:**

**Main Trial**

**Activity monitoring**

**8 days**

**AM, resting BP and Qs**

**Cannula Inserted***

**20 min**

**Baseline**

**ST1**

**6/8 min^#^**

**45 min**

**R1**

**6/8 min^#^**

**ST2**

**45 min**

**R2**

**Blood sample 1**

**Blood sample 2**

**Blood sample 3**

**Blood sample 4**

**Blood sample 5**

AM = Anthropometric measures, this included height, body mass and body fat percentage. BP = Blood Pressure; Qs = Questionnaires. *After the cannula was inserted into a suitable antecubital vein, a 3-lead ECG and non-invasive BP finger cuff were attached to the participant prior to initiation of the baseline period. #PASAT = 8 min; IAPS = 6 min. ST1 = Stress Task 1; R1 = Recovery 1; ST2 = Stress Task 2; R2 = Recovery 2. Blood pressure and heart rate were collected during the final 10 minutes of the baseline period, throughout both stress tasks, for 8 minutes immediately post each stress task and for 8 minutes at the end of each recovery period.
